# Foreground Segmentation in Depth Imagery Using Depth and Spatial Dynamic Models for Video Surveillance Applications

**DOI:** 10.3390/s140201961

**Published:** 2014-01-24

**Authors:** Carlos R. del-Blanco, Tomás Mantecón, Massimo Camplani, Fernando Jaureguizar, Luis Salgado, Narciso García

**Affiliations:** 1 Grupo de Tratamiento de Imágenes, E.T.S.I de Telecomunicación, Universidad Politécnica de Ma Avenida Complutense 30, Madrid 28040, Spain; E-Mails: tmv@gti.ssr.upm.es (T.M.); mac@gti.ssr.upm.es (M.C.); fjn@gti.ssr.upm.es (F.J.); lsa@gti.ssr.upm.es (L.S.); narciso@gti.ssr.upm.es (N.G.); 2 Video Processing and Understanding Laboratory, Universidad Autónoma de Madrid, Madrid 28049, Spain

**Keywords:** depth sensors, foreground segmentation, video surveillance, Bayesian network

## Abstract

Low-cost systems that can obtain a high-quality foreground segmentation almost independently of the existing illumination conditions for indoor environments are very desirable, especially for security and surveillance applications. In this paper, a novel foreground segmentation algorithm that uses only a Kinect depth sensor is proposed to satisfy the aforementioned system characteristics. This is achieved by combining a mixture of Gaussians-based background subtraction algorithm with a new Bayesian network that robustly predicts the foreground/background regions between consecutive time steps. The Bayesian network explicitly exploits the intrinsic characteristics of the depth data by means of two dynamic models that estimate the spatial and depth evolution of the foreground/background regions. The most remarkable contribution is the depth-based dynamic model that predicts the changes in the foreground depth distribution between consecutive time steps. This is a key difference with regard to visible imagery, where the color/gray distribution of the foreground is typically assumed to be constant. Experiments carried out on two different depth-based databases demonstrate that the proposed combination of algorithms is able to obtain a more accurate segmentation of the foreground/background than other state-of-the art approaches.

## 1. Introduction

Video surveillance in indoor environments is an active focus of research because of its high interest for the security industry [1,2]. One of the key components of a video surveillance system is the detection of objects of interest [3,4]. Background subtraction techniques allow for the segmenting of objects of interest that are moving using a static camera. The basic approach is to robustly model the background, which is ideally a static scene, and then to detect the moving objects (the foreground) by computing which image regions do not fit the background model. For this purpose, visible, infrared (IR), thermal and depth imagery can be used.

Most of the existing works have been designed to operate with visible imagery (gray or color images). State-of-the-art algorithms have achieved a great performance in the presence of challenging situations, such as changes in illumination, shadows, color camouflage and non-static background regions [5–8]. However, they cannot properly operate under low, unpredictable or no illumination conditions. This fact is a serious drawback for applications that must work in real indoor environments, such as homes, offices or public/private facilities, where lights can be just switched off or inappropriately placed in the scene, leading to a reduction in the performance of the video surveillance system. Therefore, systems based on visible imagery require an adequate illumination installation and setting to guarantee a satisfactory foreground segmentation. This fact increases the cost of the system and complicates its deployment.

Regarding the systems based on IR imagery (near-infrared spectrum, very close to the visible imagery), they have the advantage that they can work in nighttime situations [9] by illuminating the scene with infrared light. However, they require a proper infrared illumination system, which increases the cost and the complexity of the installation. In addition, they share some problems with the visible imagery, such as shadows, reflections and foreground camouflage with the background, which can decrease the quality of the segmentation results.

Systems based on thermal sensors [10–12], which operate in the mid- and long-infrared spectrum, can work independently of the existing illumination conditions in the scene and lack some of the problems that appear in visible and IR imagery (changes in illumination, shadows and reflections). However, thermal cameras are much more expensive than the other options, and their signal-to-noise ration is also worse than that of the other type of sensors.

Surveillance systems based on low-cost depth sensors, such as the Microsoft Kinect, or the ASUS Xtion, are an excellent alternative. They achieve an excellent tradeoff among the following three aspects: installation and settings, cost and quality of the segmented foreground. The installation and setting is quite simple, since they do not have any specific illumination requirements: they can work independently of the existing illumination in the indoor environment, even in total darkness, as discussed in recent reviews [13,14]. The other advantage is the low cost of the aforementioned depth sensors, which offer a satisfactory performance for the foreground segmentation task. In addition, they are becoming more and more popular for a broad range of applications, and therefore, their price is likely to decrease in the near future as a result of economies of scale. On the other hand, although Microsoft Kinect (first and second generation) and the ASUS Xtion Pro Live can acquire both color and depth imagery, the computational cost of processing both data flows (visible and depth imagery) could be quite high, requiring special and expensive hardware. This fact motivates the interest for evaluating the performance of systems that use only depth imagery to segment the foreground, which can use acquisition devices, such as the ASUS Xtion Pro or the occipital structure sensor, which have only one depth sensor. As regards the performance of the resulting foreground segmentation, depth sensors have three main advantages. The first one is that depth sensors are not affected by moving shadows and sudden illumination changes. The second advantage is that the modeling of the dynamics of foreground pixels in depth imagery is less complex (and, therefore, more feasible) than in color imagery. In color imagery, the spatio-temporal prediction of pixel values corresponding to foreground objects is very complex, depending on many factors: variations in the illumination, reflections, changes in the point of view, *etc*. However, in depth imagery, this prediction is less complex, conditioned by the physical restrictions of the movement of the foreground objects. The inclusion of this information in the foreground/background (FG/BG) modeling improves the quality of the computed FG/BG segmentation. The third advantage is the lower misclassification probability of a foreground pixel. In color imagery, this misclassification occurs when the background and foreground have similar pixel values (which is relatively frequent). However, in depth imagery, this situation is less frequent, since two phenomena must happen at the same time: the background and foreground must be spatially located very close to each other in the real world, and the data noise must be relatively high.

In spite of the aforementioned advantages of surveillance systems based on low-cost depth sensors, there are no works that perform a high-quality foreground segmentation using depth data exclusively, according to the authors' knowledge. This claim is also confirmed by a recent review [13], focused on Kinect-based applications. Nonetheless, there are several works that combine depth and visible imagery to improve the foreground segmentation results. Leens *et al.* [15] use a color camera along with a time-of-flight camera to segment objects of interest. In this work, color and depth data are independently processed by a background subtraction algorithm called Vibe [16]. The resulting foreground masks are then combined using logical operations to obtain the final foreground mask. Similarly, Camplani and Salgado [17] utilize a Microsoft Kinect sensor to acquire visible and depth imagery. Two independent background models are created using a mixture of Gaussians algorithm (MoG), which are combined by means of a weighted average that takes into account the different characteristics of each kind of imagery. A combination of color and depth data for moving object detection purposes has been presented also in [18], in which the codebook background detection algorithm presented in [19] was adapted to the joint RGBcolor and depth (RGB-D) feature space. A different approach is adopted by Clapés *et al.* [4], where a unique per pixel background model is used for visible and depth imagery, which is acquired by a Kinect sensor. The statistics of each pixel in the background model are represented by a four-dimensional Gaussian distribution that includes RGB color and depth data. One limitation is that the single Gaussian model does not allow the managing of moving background regions. Depth imagery has been also used in conjunction with infrared imagery to achieve a better performance in [20]. An MoG algorithm was used to create two independent per-pixel background models, one for the depth imagery and another for the infrared imagery. They are combined using an agreement approach to estimate the foreground. However, the performance significantly decreases when both models disagree in the FG/BG estimation.

Although the combination of multiple sources of imagery can improve the foreground segmentation, the cost, installation and complexity of the system are significantly increased. This fact motivates the interest in developing systems based only on depth sensors, which achieve a more appealing tradeoff of the system characteristics and requirements. Although a depth-based foreground segmentation is possible using some of the depth background models involved in the previous works that combine multiple kinds of imagery, the obtained segmentation results are not completely satisfactory. The main reason is that they have applied background subtraction algorithms conceived of for color imagery to depth imagery, without specifically addressing the problems of depth sensors: the limited depth range in the acquisition process, the lack of depth information in some regions due to reflections [21] and the high level of noise of the acquired depth data. Moreover, the sensor noise follows a quadratic relationship with respect to the actual depth of the objects in the scene. This characteristic is typical for the kinds of active depth sensors that are based on the triangulation principle [22], such as Kinect. Although some of the previous problems have been satisfactorily addressed by Camplani and Salgado [17], they do not fully exploit the intrinsic characteristics of the depth imagery, such as the underlying dynamics of the moving foreground objects in the depth domain.

In this paper, we propose a combination of two algorithms to obtain a high-quality foreground segmentation using only depth data information acquired by a Kinect sensor (first generation), which is ideal for security/surveillance indoor applications that have to deal with situations of low, unpredictable or no lighting. The first algorithm is the classic MoG algorithm adapted for depth imagery. The second algorithm is based on a Bayesian network, which explicitly exploits the intrinsic characteristic of the depth data. This Bayesian network is able to accurately predict the FG/BG regions between consecutive time steps using two dynamic models, which encode the spatial and depth evolution of the FG/BG regions. The most important contribution of the paper is the proposed depth-based dynamic model. Unlike the case of visible imagery, where the color/gray distribution of the foreground is assumed to be constant (at least in certain periods of time), the depth distribution can significantly change between consecutive time steps, because of the own motion of the foreground objects. As far as the authors' knowledge, there is no proposal in the literature that deals with this problem. In the Kinect-based video surveillance re-identification system presented in [23], a simple depth-based background model (based on the work of [24]) is used. However, this model neither takes into account the problems related to the depth-sensor noise, nor does it consider the different dynamics of the depth features that characterize the moving foreground objects. On the contrary, an explicit depth-based dynamic model is proposed in this paper, allowing for the prediction of the depth evolution of foreground moving objects with an arbitrary and more realistic camera setting. Another key advantage of the proposed Bayesian network is that it is able to obtain satisfactory results in distances beyond the recommended operating conditions of the Kinect sensor, extending the range of approximately 1.2 to 3.5 m [25] up to 10–12 m thanks to the adaptive processing of the Kinect sensor noise. Excellent results have been obtained using two public databases, outperforming existing state-of-the art approaches.

The organization of the paper is as follows. A general overview of the proposed FG/BG segmentation algorithm is presented in Section 2. The proposed Bayesian network for the estimation of the FG/BG probabilities using spatial and depth correlation properties is described in Section 3, along with the applied approximate inference technique. The results obtained from testing the proposed foreground segmentation algorithm with two public databases are presented in Section 4. Finally, conclusions are drawn in Section 5.

## 2. System Description

**Figure 1.**
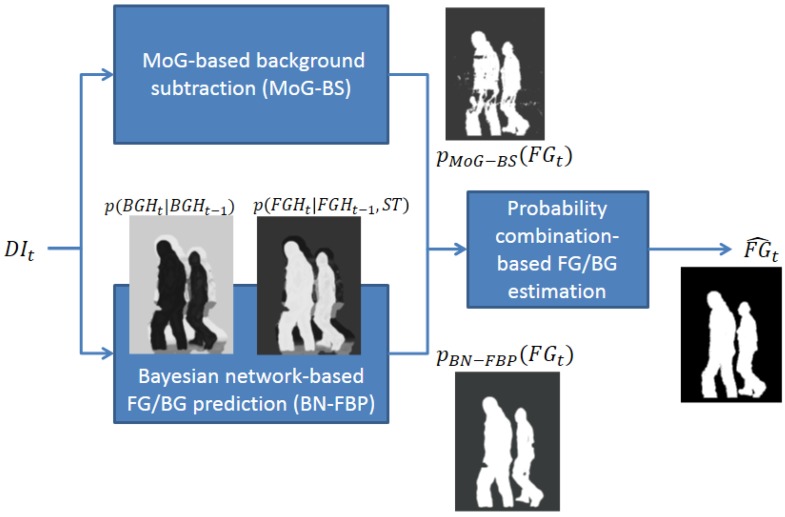
Modules of the proposed foreground/background (FG/BG) segmentation system. MoG, mixture of Gaussians.

The proposed FG/BG segmentation system consists of three modules (see Figure 1): (1) MoG-based background subtraction (MoG-BS); (2) Bayesian network-based FG/BG prediction (BN-FBP); and (3) probability combination-based FG/BG estimation. The first two modules compute a probability map of the FG/BG regions for each time step considering different and complementary approaches: the first module uses a pixel-wise strategy, whereas the second module uses a region-wise strategy. The third module combines the previous two FG/BG probability maps to estimate the final FG/BG segmentation.

The MoG-BS module is based on the algorithm presented in [17], which builds a pixel-wise probabilistic background model using an independent mixture of Gaussian distribution per pixel. This model is then used to compute for every time step, *t*, the probability of each pixel in the current depth image, *DI_t_*, to be the foreground or background. Defining *FG_t_* as the FG/BG image segmentation in the time step, *t*, where *FG_t_*(*x*) = 1 indicates that the pixel of coordinates, *x*, is the foreground and *FG_t_*(*x*) = 0 is the background, the probability of every pixel of being the foreground and background according to the MoG-BS module is represented by ***P**_MoG–BS_*(*FG_t_*(*x*) = 1) and ***P**_MoG–BS_*(*FG_t_*(*x*) = 0), respectively. The parameters of the involved models are automatically adapted to the distance-dependent noise that affects the Kinect-based depth data (see [22]). The computed pixel-wise probabilistic background model is also used to obtain a depth-based representation of the most probable background at each time step, *DBG_t_*, which will be used by the BN-FBP module. This is carried out by selecting for each pixel the mean value of the most probable Gaussian (*i.e.*, the Gaussian with the highest weight) in the mixture.

The BN-FBP module also estimates per pixel probabilities of FG/BG, but using a region-based approach that exploits the spatial and depth correlations of the FG/BG regions in depth imagery across time. The probability of every pixel of being the foreground and background according to this module is represented by ***P**_BN–FBP_*(*FG_t_*(*x*) = 1) and ***P**_BN–FBP_*(*FG_t_*(*x*) = 0), respectively. The BN-FBP module is the real innovation of this paper, and it is described in detail in Section 3.

The third and last module computes the FG/BG segmentation by combining the pixel-wise probabilities, ***P**_MoG–BS_*(*FG_t_*(*x*)) and ***P**_BN–FBP_*(*FG_t_*(*x*)). First, the combined FG/BG probabilities are obtained through the expression ***P**_comb_*(*FG_t_*(*x*)) = ***P**_MoG–BS_*(*FG_t_*(*x*))***P**_BN–FBP_*(*FG_t_*(*x*)), which, from a probabilistic point of view, represents the union of the following two events [26]: that the pixel of coordinates, *x*, is considered as the foreground by both the BN-FBP and BN-FBP modules with a high probability. This approach minimizes the number of false positives, since both modules must agree to consider a pixel as the foreground. Finally, the FG/BG binary segmentation of every pixel, ${\hat{FG}}_{t}(x)$, is obtained by selecting the foreground or background event of higher probability: ***P**_comb_*(*FG_t_*(*x*) = 1) and ***P**_comb_*(*FG_t_*(*x*) = 0), respectively.

## 3. BN-FBP Module

A description of the proposed Bayesian network for the estimation of the FG/BG probabilities is presented in Section 3.1. The derivation of the posterior joint probability density function (pdf) related to the Bayesian network is presented in Section 3.2. The spatial and depth dynamic models involved in the derivation of the previous posterior joint pdf are described in Sections 3.3 and 3.4. Lastly, the process of inference is explained in Section 3.6, which is used to obtain an accurate approximation of the posterior joint pdf and, thus, the desired FG/BG probabilities.

### 3.1. Description of the Bayesian Network

**Figure 2.**
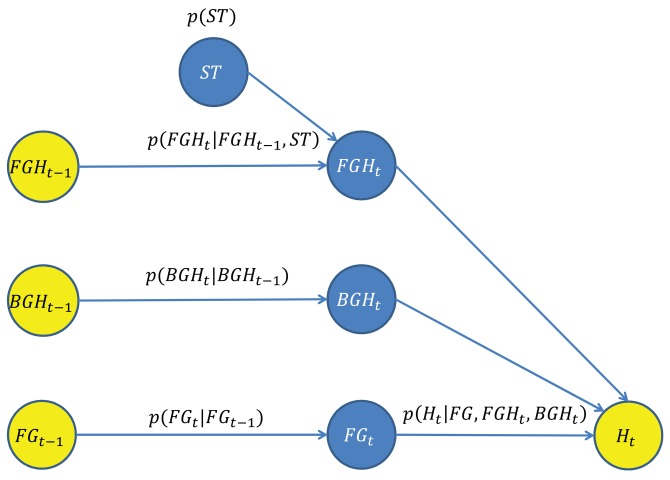
Proposed Bayesian network for the estimation of the FG/BG probabilities.

A novel Bayesian network for the estimation of the FG/BG probabilities has been designed, which takes advantage of the spatial and depth correlation properties of the depth imagery across time. The goal is to estimate ***P**_BN–FBP_*(*FG_t_*(*x*)), the FG/BG probabilities for every pixel and time step, using the Bayesian network shown in Figure 2, which is composed by a set of nodes (variables) and directed edges (relationships between variables). Blue nodes represent unknown variables to be estimated, and yellow nodes represent observed variables used as input data.

The variable *FG_t_* = {*FG_t_*(*x*)∣*x* ∈ *S_DI_*} is the FG/BG image segmentation at time step *t*, where *S_DI_* is the set of pixel coordinates of the depth image. The variable, *FG_t_*, depends on both variables, *FG_t_*_−1_ and *H_t_*.

The variable, *FG_t_*_−1_, is the estimated FG/BG image segmentation at the previous time step. The relationship between *FG_t_* and *FG_t_*_−1_ is determined by a spatial dynamic model described in Section 3.3, which predicts the location of the foreground regions at the current time step.

The variable *H_t_* = {*H_t_*(*x*)∣*x* ∈ *S_DI_*} is the set of observations/measurements taken from the current depth image, *DI_t_*. Every individual observation, *H_t_*(*x*), is a local depth histogram of a region of *DI_t_* that encodes its depth value distribution. For this purpose, squared regions of side *l* and centered on every *x* are considered. The relationship between *FG_t_* and *H_t_* is determined by an observation model described in Section 3.5, which evaluates the degree of agreement/fitting among a candidate FG/BG segmentation, *FG_t_*, the observations, *H_t_*, and the predicted depth-based appearance of the FG/BG regions at the current time step.

The predicted depth-based appearance of the FG/BG regions involved in the above observation model are represented by the variables, *FGH_t_* and *BGH_t_*, respectively. The depth-based appearance is modeled by a bag of features' representation [27]. Thus, the foreground appearance at time step *t*, *FGH_t_*, is represented by a set of unordered local depth histograms computed from the foreground regions *FG_t_*(*x*) = 1. The computation of the local depth histograms is the same as for *H_t_*(*x*). Likewise, the background appearance, *BGH_t_*, is computed from the background regions *FG_t_*(*x*) = 0. However, direct computation of *FGH_t_* and *BGH_t_* is not possible, since the FG/BG regions at the current time step are unknown. Therefore, *FGH_t_* and *BGH_t_* are estimated by predicting the appearance evolution of *FGH_t_*_−1_ and *BGH_t_*_−1_, which can be computed from the available FG/BG segmentation at the previous time step, ${\hat{FG}}_{t-1}(x)$.

The estimation of *FGH_t_* is obtained from *FGH_t_*_−1_ using a depth-based dynamic model for foreground regions described in Section 3.4. The variable, *FGH_t_*_−1_, is obtained by computing the local depth histograms from the regions of *DI_t_*_−1_ that were segmented as the foreground at the previous time step $\left({\hat{FG}}_{t-1}(x)=1\right)$. The proposed depth-based dynamic model also depends on the variable, *ST*, that encodes the expected shifts in the depth of foreground regions between consecutive time steps.

Similarly to the estimation of *FGH_t_*, the estimation of *BGH_t_* is obtained from *BGH_t_*_−1_ using a depth-based dynamic model for background regions described in Section 3.4. The variable, *BGH_t_*_−1_, is obtained by computing the local depth histograms from the regions of *DBG_t_* (the depth-based representation of the most probable background obtained from the MoG-BS module). The advantage of using *DBG_t_* (instead of the background regions of *DI_t_*_−1_) is that a more accurate background appearance model is obtained, since *DBG_t_* can be considered as a temporal filtered version of the background regions of *DI_t_*.

Table 1 shows a summary of all variables and the main parameters involved in the Bayesian network.

### 3.2. Derivation of the Posterior Joint pdf

From a Bayesian perspective, the goal is to estimate the posterior joint pdf, ***P***(*FG_t_, FGH_t_, BGH_t_, ST∣FG_t_*_−1_, *FGH_t_*_−1_, *BGH_t_*_−1_, *H_t_*), given the set of observed variables, {*FG_t_*_−1_, *FGH_t_*_−1_, *BGH_t_*_−1_, *H_t_*}. The posterior joint pdf can be derived using the chain rule for Bayesian networks as:

(1) $$\begin{array}{c} P(FG_{t},FGH_{t},BGH_{t},ST|FG_{t-1},FGH_{t-1},BGH_{t-1},H_{t})=\\ \frac{P(FG_{t},FGH_{t},BGH_{t},ST,FG_{t-1},FGH_{t-1},BGH_{t-1},H_{t})}{P(FG_{t-1},FGH_{t-1},BGH_{t-1},H_{t})}=\\ \frac{P(H_{t}|FG_{t},FGH_{t},BGH_{t})P(FG_{t}|FG_{t-1})P(FGH_{t}|FGH_{t-1},ST)P(ST)P(BGH_{t}|BGH_{t-1})}{P(FG_{t-1},FGH_{t-1},BGH_{t-1},H_{t})} \end{array}$$

where ***P***(*FG_t_*_−1_,*FGH_t_*_−1_,*BGH_t_*_−1_,*H_t_*) is just a normalization constant given by:

(2) $$\begin{array}{c} P(FG_{t-1},FGH_{t-1},BGH_{t-1},H_{t})=\\ ⨌P(FG_{t},FGH_{t},BGH_{t},ST,FG_{t-1},FGH_{t-1},BGH_{t-1},H_{t})\text{d}FG_{t}\text{d}FGH_{t}\text{d}BGH_{t}\text{d}ST=\\ ⨌P(H_{t}|FG_{t},FGH_{t},BGH_{t})P(FG_{t}|FG_{t-1})P(FGH_{t}|FGH_{t-1},ST)\cdot \\ P(ST)P(BGH_{t}|BGH_{t-1})\text{d}FG_{t}\text{d}FGH_{t}\text{d}BGH_{t}\text{d}ST \end{array}$$

The probability term, ***P***(*FG_t_*∣*FG_t_*_−1_), encodes the prior knowledge about what regions could be FG/BG given the previous FG/BG estimation. Its expression is defined by a spatial dynamic model described in Section 3.3.

The probability terms, ***P***(*FGH_t_*∣*FGH_t_*_−1_, *ST*) and *P*(*ST*), predict the depth-based appearance of the foreground regions between consecutive time steps. Similarly, the probability term, ***P***(*BGH_t_*∣*BGH_t_*_−1_), predicts the depth-based appearance of the background regions between consecutive time steps. The dynamic models involved in the prediction of depth-based FG/BG appearances are described in Section 3.4.

The last probability term, ***P***(*H_t_*∣*FG_t_, FGH_t_, BGH_t_*), evaluates the degree of agreement/coherence between the depth data in the current depth image, *DI_t_*, and a set of hypothetical values of {*FG_t_, FGH_t_, BGH_t_*}, which define the FG/BG spatial distribution and the FG/BG appearance. The observation model used to compute the above pdf is described in Section 3.5.

Finally, the desired FG/BG probability, ***P**_BN–FBP_*(*FG_t_*) (the output of the BN-FBP module), is obtained by marginalizing out the variables, {*FGH_t_, BGH_t_, ST*}, from the posterior joint pdf as:

(3) $$\begin{array}{c} P_{BN-\text{FBP}}(FG_{t})=P(FG_{t}|FG_{t-1},FGH_{t-1},BGH_{t-1},H_{t})=\\ ∭P(FG_{t},FGH_{t},BGH_{t},ST|FG_{t-1},FGH_{t-1},BGH_{t-1},H_{t})\text{d}FGH_{t}\text{d}BGH_{t}\text{d}ST \end{array}$$

### 3.3. Spatial Dynamic Models

The spatial dynamic model for the foreground regions is based on a proximity concept: the neighborhood of the foreground regions at *t* − 1 is likely to be the foreground at *t*. This model is justified by the fact that typically, foreground regions between consecutive time steps are spatially overlapped, which is particularly true for the following operating conditions: an acquisition rate of 30 frames per second (maximum frame rate of the Kinect sensor) and the specific dynamics of the objects that can appear in indoor scenes (people, animals, *etc*.). Based on the above spatial dynamic model for the foreground and considering a particular pixel of coordinates, *x*, the expression of the foreground spatial prior pdf is given by:

(4) $$P(FG_{t}(x)=1|FG_{t-1})=\max\{N(x;x_{FG_{t-1}},\Sigma_{\text{spa}})|x_{FG_{t-1}}\in S_{FG_{t-1}}\}$$

where *S*_*FG*_*t*−1__ is the set of pixels segmented as the foreground at *t* − 1 and *N*(*x*; *x*_*FG*_*t*−1__,Σ*_spa_*) is a Gaussian function of mean *x*_*FG*_*t*−1__ and covariance matrix Σ*_spa_*. The parameter, Σ*_spa_*, controls the expected spatial displacements of the foreground regions, which is assumed to be isotropic, since there is no information about the velocity or the acceleration. According to the above expression, the foreground spatial pdf of one pixel depends on the closest pixel segmented as the foreground at the previous time step. For this reason, the maximum of the Gaussian contributions has been used instead of the mean of them. This approach allows for a more uniform behavior of the expected foreground regions, minimizing the differences in probability among pixels that were fully and only partially surrounded by foreground pixels at the previous time step.

On the other hand, the background spatial prior pdf of one pixel is just the complementary value of the foreground one:

(5) $$P(FG_{t}(x)=0|FG_{t-1})=1-P(FG_{t}(x)=1|FG_{t-1})$$

Given the FG/BG spatial prior pdf of every pixel, the FG/BG prior pdf of the whole image is computed as:

(6) $$P(FG_{t}|FG_{t-1})=\underset{x\in S_{DI}}{\text{∏}}P(FG_{t}(x)|FG_{t-1})$$

where *S_DI_* is the set of pixel coordinates in the depth image.

### 3.4. Depth-Based Appearance Dynamic Models

The depth-based appearance dynamic model for the foreground regions is based on the following concept: the depth values of foreground regions between two consecutive time steps are assumed to be close to each other. Thus, the prediction of the appearance of a foreground region, represented by a depth histogram, *FGH_t_*(*i*) ∈ *FGH_t_*, between consecutive time steps can be expressed as:

(7) $$P(FGH_{t}(i)|FGH_{t-1}(i),ST)=N(FGH_{t}(i);\text{shift}(FGH_{t-1}(i),ST),\Sigma_{\text{FGH}})$$

where *N*(*FGH_t_*(*i*); shift(*FGH_t_*_−1_(*i*), *ST*), Σ*_FGH_*) is a Gaussian of the mean output of the function, shift(*FGH_t_*_−1_(*i*), *ST*), and covariance Σ*_FGH_* The function, shift(*FGH_t_*_−1_(*i*), *ST*), represents a shifting of magnitude, *ST*, in the histogram, *FGH_t_*_−1_(*i*). This amounts to a linear displacement of value *ST* in the depth values of the foreground region, where the depth histogram, *FGH_t_*_−1_(*i*), was computed. The covariance matrix, Σ*_FGH_*, encodes the uncertainty of the proposed linear model, due to the deformable nature of the foreground objects.

On the other hand, the expected depth displacement of a foreground region is modeled by the Gaussian of zero mean:

(8) $$P(ST)=N(ST;0,\sigma_{ST}^{2})$$

where the variance, $\sigma_{ST}^{2}$, defines a range of probable depth displacements for the foreground regions between consecutive time steps.

The prediction of the appearance of the whole foreground, represented by the set of depth histograms, *FGH_t_*, is given by:

(9) $$P(FGH_{t}|FGH_{t-1},ST)=\underset{i}{\text{∏}}P(FGH_{t}(i)|FGH_{t-1}(i),ST)$$

The prediction of the appearance of the background, represented by the set of depth histograms, *BGH_t_*, is similar to that of the foreground, but without considering any depth displacement, since the background is considered static or quasi-static. Its expression is given by:

(10) $$P(BGH_{t}|BGH_{t-1})=\underset{j}{\text{∏}}P(BGH_{t}(j)|BGH_{t-1}(j))$$

where *BGH_t_*(*j*) ∈ *BGH_t_* and:

(11) $$P(BGH_{t}(j)|BGH_{t-1}(j))=N(BGH_{t}(j);BGH_{t-1}(j),\Sigma_{\text{BGH}})$$

where Σ*_BGH_* is used to model smooth variations in depth of the background regions due to quasi-static backgrounds.

### 3.5. Depth-Based Observation Model

The observation model evaluates the degree of agreement/coherence between the set of depth histograms, *H_t_*, computed from the current depth image, *DI_t_*, and the predicted depth-based appearance of the FG/BG regions, given by the variables, *FG_t_*, *FGH_t_* and *BGH_t_*. The implementation of the observation model is based on a discriminative approach that uses an on-line trained logistic regression classifier to compute ***P***(*H_t_*(*x*)∣*FG_t_*(*x*), *FGH_t_, BGH_t_*), the probability that a depth histogram, *H_t_*(*x*) ∈ *H_t_* (computed from a region of *DI_t_* centered at *x*), is the foreground (*FG_t_*(*x*) = 1) or background (*FG_t_*(*x*) = 0), given the predictions about the foreground and background depth-based appearances (*FGH_t_* and *BGH_t_*, respectively). The cost function used by the logistic regression classifier is:

(12) $$J(\theta )=\frac{1}{N_{H}}\overset{N_{H}}{\underset{i=1}{\text{∑}}}\left[-y(i)\log({\text{sg}}_{\theta }(x(i)))-(1-y(i))\log(1-\text{s}{\text{g}}_{\theta }(x(i)))\right]$$

The variable, *x*(*i*) ∈ *FGH_t_* ∪ *BGH_t_*, is a depth histogram used for the classifier training, and *y*(*i*) is its associated label indicating to which class (FG/BG) it belongs. The variable, *N_H_*, is the total number of training data, which is equal to the number of elements in the set union, *FGH_t_* ∪ *BGH_t_*. The vector, *θ*, defines a hyperplane that splits the feature space into the two considered classes, and it is calculated by minimizing the above cost function using the gradient descendent algorithm. Additionally, sg*_θ_*(*x*(*i*)) is the sigmoid function, which is used as much as in the computation of the optimal *θ* in the training stage as in the classification of every depth histogram, *H_t_*(*x*), in the evaluation stage. Note that the Kinect noise distribution is internally managed by the classifier, since the training data (the predicted FG/BG depth histograms) are obtained from the noisy FG/BG regions of *DI_t_*_−1_.

Finally, the pdf, ***P***(*H_t_*∣*FG_t_, FGH_t_, BGH_t_*), that takes into account the whole set of depth histograms, *H_t_*, is computed as:

(13) $$P(H_{t}|FG_{t},FGH_{t},BGH_{t})=\underset{x\in S_{DI}}{\text{∏}}P(H_{t}(x)|FG_{t}(x),FGH_{t},BGH_{t})$$

where *S_DI_* has been already defined as the set of pixel coordinates in the depth image, *DI_t_*.

### 3.6. Inference

The expression of the posterior joint pdf, ***P***(*FG_t_, FGH_t_, BGH_t_, ST*∣*FG_t_*_−1_,*FGH_t_*_−1_,*BGH_t_*_−1_,*H_t_*), cannot be analytically determined due to the non-linear and multi-modal nature of the FG/BG probability estimation problem. Therefore, an approximate inference approach is used to obtain an estimation via the process of Rao–Blackwellization [28], which is able to compute an accurate estimation in the proposed high dimensional Bayesian network. This process samples some of the variables using a hierarchical sampling strategy [29] and marginalizes out the other ones in an exact way using a grid-based method [30]. Thus, the process of inference is divided into two parts, where one of them can be computed exactly and, therefore, achieving an estimation with less variance. The posterior joint pdf is Rao–Blackwellized as:

(14) $$P(FG_{t},FGH_{t},BGH_{t},ST|FG_{t-1},FGH_{t-1},BGH_{t-1},H_{t})\propto P_{\text{GRID}}(FG_{t})P_{\text{HIER}}(FGH_{t},BGH_{t},ST)$$

where:

(15) $$P_{\text{HIER}}(FGH_{t},BGH_{t},ST)=P(FGH_{t}|FGH_{t-1},ST)P(ST)P(BGH_{t}|BGH_{t-1})$$

(16) $$P_{\text{GRID}}(FG_{t})=P(H_{t}|FG_{t},FGH_{t},BGH_{t})P(FG_{t}|FG_{t-1})$$

The probability term, ***P**_HIER_*(*FGH_t_, BGH_t_, ST*), is approximated by a set of *N_p_* samples {*FGH_t_*^(*p*)^, *BGH_t_*^(*p*)^, *ST*^(*p*)^∣*p* = 1,…, *N_p_*} as:

(17) $$P_{\text{HIER}}(FGH_{t},BGH_{t},ST)=\overset{N_{p}}{\underset{p=1}{\text{∑}}}\delta (FGH_{t}-FGH_{t}^{\left(p\right)},BGH_{t}-BGH_{t}^{\left(p\right)},ST-ST^{\left(p\right)})$$

where *δ*(*x*) is the Dirac delta function. The samples are drawn through a hierarchical sampling strategy described in Algorithm 1.

**Algorithm 1** Hierarchical sampling of the variables, {*FGH_t_, BGH_t_, ST*}.
For *p*=1 to *N_p_*:(1)Draw a sample, *ST*^(*p*)^, from 
$P(ST)=N(ST;0,\sigma_{ST}^{2})$;(2)Conditioned on *ST*^(*p*)^, draw a sample, 
$FGH_{t}^{\left(p\right)}$, from ***P***(*FGH_t_*∣*FGH_t_*_−1_,*ST*^(*p*)^) = ∏*_i_**P*** (*FGH_t_*(*i*)∣*FGH_t_*_−1_(*i*),*ST*^(*p*)^);(3)Draw a sam ple, 
$BGH_{t}^{\left(p\right)}$, from ***P***(*BGH_t_*∣*BGH_t_*_−1_) = ∏*_j_**P***(*BGH_t_*(*j*)∣*BGH_t_*_−1_(*j*)).End.


Conditioned on a drawn joint sample, $\left\{FGH_{t}^{\left(p\right)},BGH_{t}^{\left(p\right)}\right\}$, and taking into account the discrete nature of *FG_t_*, the probability term, ***P**_GRID_*(*FG_t_*), can be expressed as:

(18) $$\begin{array}{c} P_{\text{GRID}}(FG_{t})=P(H_{t}|FG_{t},FGH_{t}^{\left(p\right)},BGH_{t}^{\left(p\right)})P(FG_{t}|FG_{t-1})=\\ \overset{N_{H}}{\underset{i=1}{\text{∏}}}P(H_{t}(i)|FG_{t}(i),FGH_{t}^{\left(p\right)},BGH_{t}^{\left(p\right)})P(FG_{t}(i)|FG_{t-1}) \end{array}$$

which can be exactly estimated using a grid-based method that computes a weight, *w*(*i*), for each binary state (FG/BG) of $FG_{t}^{\left(q\right)}(i)$ as:

(19) $$P_{\text{GRID}}(FG_{t})=\overset{N_{H}}{\underset{i=1}{\text{∏}}}\overset{1}{\underset{q=0}{\text{∑}}}w{\left(i\right)}^{\left(p)(q\right)}\delta (FG_{t}(i)-FG_{t}^{\left(q\right)}(i))$$

where every weight, *w*(*i*)^(^*^p^*^)(^*^q^*^)^, is computed as:

(20) $$w{\left(i\right)}^{\left(p)(q\right)}=P(H_{t}(i)|FG_{t}^{\left(q\right)}(i),FGH_{t}^{\left(p\right)},BGH_{t}^{\left(p\right)})P(FG_{t}^{\left(q\right)}(i)|FG_{t-1})$$

where the expression of each involved probability term has been already defined in Section 3.

As a result, the posterior joint pdf can be expressed as:

(21) $$\begin{array}{l} P(FG_{t},FGH_{t},BGH_{t},ST|FG_{t-1},FGH_{t-1},BGH_{t-1},H_{t})\propto \\ \overset{N_{p}}{\underset{p=1}{\text{∑}}}\overset{N_{H}}{\underset{i=1}{\text{∏}}}\overset{1}{\underset{q=0}{\text{∑}}}w{\left(i\right)}^{\left(p)(q\right)}\delta (FG_{t}(i)-FG_{t}^{\left(q\right)}(i))\delta (FGH_{t}-FGH_{t}^{\left(p\right)},BGH_{t}-BGH_{t}^{\left(p\right)},ST-ST^{\left(p\right)}) \end{array}$$

This expression is then normalized to one to be a correctly defined probability.

Finally, the desired FG/BG probabilities, ***P**_BN–FBP_*(*FG_t_*) (the output of the BN-FBP module), are estimated by marginalizing out the variables, {*FGH_t_, BGH_t_, ST*}, from the above expression as:

(22) $$\begin{array}{c} P_{BN-\text{FBP}}(FG_{t})=P(FG_{t}|FG_{t-1},FGH_{t-1},BGH_{t-1},H_{t})=\\ \overset{N_{p}}{\underset{P=1}{\text{∑}}}P(FG_{t},FGH_{t}^{\left(p\right)},BGH_{t}^{\left(p\right)},ST^{\left(p\right)}|FG_{t-1},FGH_{t-1},BGH_{t-1},H_{t}) \end{array}$$

## 4. Results

The proposed FG/BG segmentation system is tested and compared with other state-of-the-art algorithms using two different depth-based datasets. The first one was presented in [31]. This dataset is composed by three different indoor sequences acquired by Kinect sensors. The dataset contains depth and RGB data, although only the depth information is used here to emulate the situation where the illumination conditions are so poor that it is not reliable to use color imagery. The sequences represent a real example of a surveillance application with a set of challenging problems, such as: lack of depth information for large image regions (due to reflections and out-of-range data), crowded situations, high level of noise in the acquired depth data (as the Kinect sensor is operating far beyond the recommended operating settings to cover a large surveillance area) and mutual interference of the Kinect devices (they are active sensors that emit structured light). For this paper, the ground truth, composed by foreground silhouettes, has been manually generated; more specifically one ground truth image has been created for every five frames. The second dataset [32] is composed by four sequences also acquired by two Kinect cameras, which are located at the same position, but pointing at opposite directions. Color (not used by our system), depth and ground truth information are available for each sequence, containing over 40 different people per sequence.

The metrics used to perform the evaluation of the algorithms are: false positive rate (FPR), which represents the fraction of background pixels that are incorrectly marked as foreground; false negative rate (FNR), which represents the fraction of foreground pixels that are incorrectly marked as background; total error (TE), which represents the total number of misclassified pixels normalized with respect to the image size; and one similarity measure, *S* (known as Jaccard's index [33]), which combines the FPR and FNR information as:

(23) $$S(A,B)=\frac{A\cap B}{A\cup B}$$

where the set, *A*, is the region segmented by the algorithm, and the set, *B*, is the region corresponding to the ground truth. The similarity measure, *S*, can have any value between zero and one; values close to one indicate that *A* and *B* are very similar, and values close to zero indicate that *A* and *B* are completely different.

To rank the accuracy of the analyzed methods, the overall metric proposed in [34] has been used. Let us define rank*_i_*(*m*, *sq*) as the rank of the *i*–*th* method for the performance metric, *m*, in the sequence, *sq*, then, the average ranking of the method, *i*, in the sequence, *sq*, is calculated as:

(24) $$RM_{i}=\frac{1}{N_{m}}\sum_{m}{\text{rank}}_{i}(m,sq)$$

where *N_m_* is the number of performance metrics.

The final overall metric, *RC*, is computed combining the performance across different metrics and sequences into a single rank, indicating the global performance of one method with respect to the others. The global ranking of the *i*–*th* method, *RC_i_*, is computed as:

(25) $$RC_{i}=\frac{1}{N_{sq}}\sum_{sq}RM_{i}$$

where *N_sq_* is the number of sequences.

The performance of the proposed method, referred to from now on as BayesNet, is compared with other state-of-the-art background subtraction techniques: pixel-based adaptive segmenter(PBAS) [35], Vibe [16], self-organizing map foreground segmentation (SOM) [36], adaptive-Gaussian-mixture-model foreground segmentation (*MoG_Ziυ_*) [24], and depth-mixture-of-Gaussian foreground segmentation (*MoG_D_*) [17]. Some of these techniques were deeply evaluated in the “Workshop on Change Detection 2012” [34]. In particular, the non-parametric foreground segmentation method, PBAS, has obtained outstanding results with respect to other approaches. This method models the background using the recent history of observed pixel values. Then, a decision threshold computed dynamically for each pixel is used to determine if it belongs to the foreground. Vibe is another non-parametric technique that models the background by using the information of the past pixels at the same position and some pixels in the neighborhood. In every time step, the update of the background model is partially performed by randomly selecting some pixels in the neighborhood without considering their insertion time in the model. SOM is a neural network-based approach that uses the self-organizing map method to detect the foreground objects. One advantage of this approach is that it does not make any assumption about the pixel distribution. The strategy to update the network weights is called *winner takes all*. *MoG_Ziυ_* is an efficient adaptive algorithm that uses a Gaussian Mixture Model to perform the background subtraction, which also automatically computes the number of Gaussian components for each pixel. This method was also recently used in a depth-based surveillance system for re-identification tasks proposed in [23]. The last method, *MoG_D_*, is a depth-based background subtraction method based on a mixture of Gaussians, which was originally used in combination with other color-based background subtraction strategies to perform the foreground segmentation. *MoG_D_* is the only method that has been designed taking into account some specific properties of the depth imagery (adaptive processing of noise), which is also used in the proposed framework in the MoG-BS module. Therefore, this algorithm can be viewed as a baseline method to measure the performance increase with regard to the proposed method. The other techniques were devised to use color imagery, and therefore, they had to be adapted to use only depth information for the present comparative evaluation. The reason for this decision is that there are no techniques in the literature that exclusively use depth information for the task of foreground segmentation, since the existing works use the depth in combination with color or infrared imagery, focusing more in the fusion of data than in the exploitation of the depth characteristics.

For the comparison, the following parameters have been used for the BayesNet algorithm: *l* = 17 pixels, $\Sigma_{\text{spa}}=\frac{17}{2}I$ (where *I* is the identity matrix), the number of bins used for the *H_t_*(*x*), *FGH_t_*(*x*) and *BGH_t_*(*x*) histograms is 256, Σ*_FGH_* = 64*I*, Σ*_BGH_* = 16*I* and *N_p_* = 200 particles. Regarding the depth histogram computation in the BN-FBP module, pixels with zero value (indicating a lack of depth information) are filtered, since it is assumed that a foreground object cannot be composed by this special value. Although this assumption could not be totally true for some situations (foreground objects with reflecting regions, foreground objects out of the dynamic range of the sensor), it tends to produce better FG/BG segmentation, since *a priori*, it is not possible to distinguish if a zero value comes from a foreground or a background region.

Regarding the setting of the parameters used by the other algorithms, the following strategy has been adopted. Initially, the parameters that the authors selected in their original papers as optimal have been used. Furthermore, other configurations have been taken into account, which were found to be optimal in reviews or other works containing comparisons among algorithms (as in the case of the change detection challenge). Later, those parameters have been refined to maximize the *S* metric, which is a single parameter that combines the impact of the false positives and negatives. Therefore, the best combination of parameters has been used for each algorithm in order to maximize the *S* metric. On the other hand, with the purpose of making as fair as possible the comparison among the different algorithms, we have homogenized some implementation criteria. The fist one refers to the different dynamic range of color and depth imagery. In this sense, the same number of bins for the depth histograms, 256 bins, has been used by means of the quantization of the higher dynamic range of the depth imagery. This limitation comes because the original implementation of almost all the algorithms are thought to work only with color/gray imagery with a resolution of eight bits per channel. In addition, there is no source code available for some of them (only an executable, where the user can change the parameters), and therefore, it is not possible to adapt the number of bins. Another implementation criterion is that the special zero value (indicating no depth data) in the depth imagery has been considered as a possible value in all the algorithms.

The results of the different algorithms using the *lobby*1 sequence in the dataset [31] are presented in Table 2. The proposed technique, BayesNet, achieves the best performance on average (*RM*value) and also achieves the lowest total error (TE) and the highest similarity, *S*. Although BayesNet has the second best performance score in the FPR and FNR metrics, it has the best balance between both types of errors, as the TE, *S* and *RM* metrics demonstrate.

**Figure 3.**
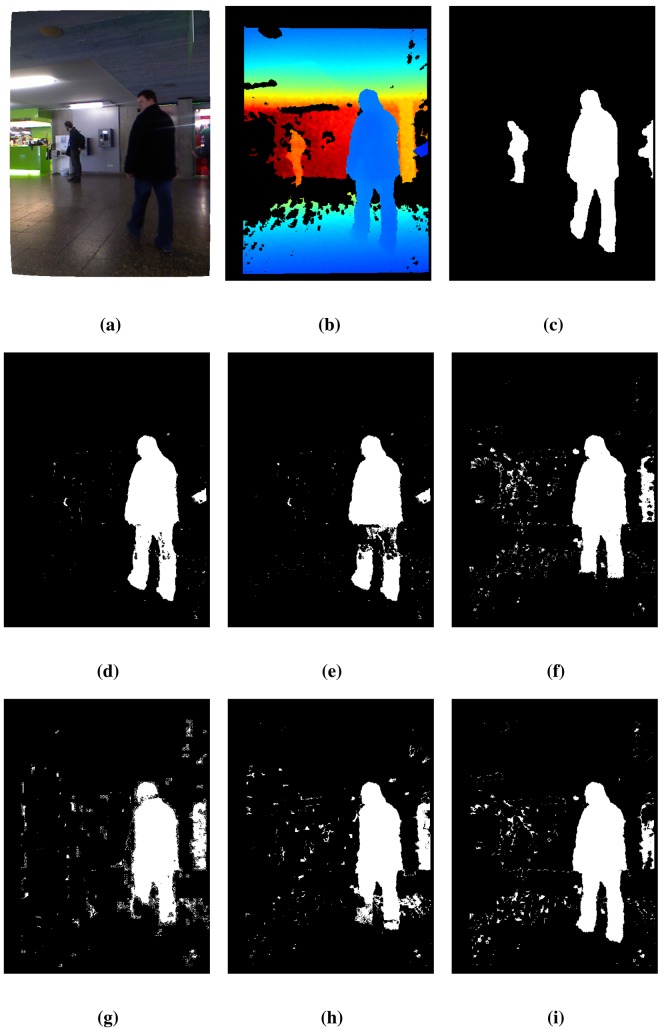
Results for frame 420 of the *lobby*1 sequence. See the text for a detailed explanation. (**a**) Color; (**b**) Depth; (**c**) Ground truth; (**d**) BayesNet; (**e**) *MoG_D_*; (**f**) PBAS; (**g**) Vibe; (**h**) SOM; (**i**) *MoG_Ziυ_*.

Figure 3 shows some details of the data provided by the Kinect cameras (color and depth data) for the *lobby*1 sequence along with the results of the FG/BG segmentation. Figure 3a shows the 420 RGB frame. Figure 3b shows the corresponding depth image, which has large areas of pixels without depth information (marked in black). Some of these areas arise from the calibration process used to align the color and depth imagery, which is needed to validate the ground truth, since it has been performed over the color imagery. Figure 3c contains the ground truth. Additionally, Figure 3d–i shows the segmentation results for the proposed method, BayesNet, and the algorithms, *MoG_D_*, PBAS, Vibe, SOM and *MoG_Ziυ_*. As can be observed, the proposed method, BayesNet, and *MoG_Ziυ_* offer the best results according to the ground truth, although BayesNet achieves a significantly smaller number of false positives. The other algorithms have a lower performance in terms of segmentation accuracy, false positives and false negatives. Note that there is a person who has not been detected by any algorithm, since that person has been still for a long period of time, and therefore, he has been considered as part of the background.

Table 3 presents the detection results using the *lobby*2 sequence of the dataset [31]. The results show the same trend as those of Table 2: the proposed technique, BayesNet, achieves the best results on average, indicated by the *RM* value, and also for the TE and *S* metrics, while for the FNR and FPR metrics, it achieves the second best performance.

**Figure 4.**
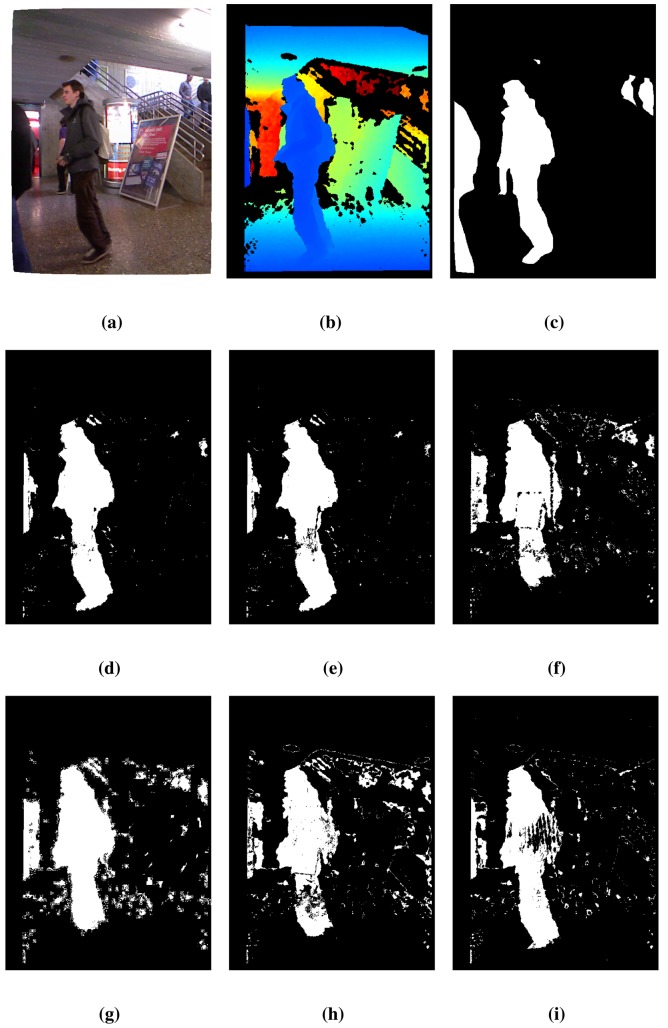
Results for frame 410 of the *lobby*2 sequence. See the text for a detailed explanation. (**a**) Color; (**b**) Depth; (**c**) Ground truth; (**d**) BayesNet; (**e**) *MoG_D_*; (**f**) PBAS; (**g**) Vibe; (**h**) SOM; (**i**) *MoG_Ziυ_*.

Some qualitative FG/BG segmentation results are shown in Figure 4 for the 410 frame of the sequence, *lobby*2, which follow the same arrangement as in Figure 3. The proposed method, BayesNet, and the *MoG_D_* algorithm fit quite well with the ground truth, but BayesNet has a slightly better performance in the number of false negatives (reduces the holes inside the foreground). The other techniques have worse performance in terms of segmentation accuracy, false positives and false negatives. Note that there are two people at the end of the scene that are barely detected for any algorithm, since they are out of the range of the Kinect sensor.

Table 4 shows the foreground segmentation results using the *lobby*3 sequence of the dataset [31]. In this case, BayesNet and PBAS share the best ranking on average (*RM*) and also for the similarity metric, *S*. However, BayesNet is better in the TE metric than PBAS (in fact, it is the best). Regarding the FNR and FPR metrics, BayesNet, without being the best, achieves one of the best scores.

Table 5 shows the FG/BG segmentation results using the *cam*1 sequence of the dataset [32]. In this case, *BayesNet* has the best ranking on average (*RM*) and also achieves the best results for the metrics, *S*, TE and FNR. Only, it is slightly outperformed by the PBAS algorithm in the FPR metrics. Nonetheless, BayesNet achieves the best global results indicated by the *RM* and *S* metrics.

Similarly, Table 6 shows the FG/BG segmentation results using the *cam*2 sequence of the dataset [32]. Following the same trend as in the previous case, BayesNet achieves the best ranking in average (*RM*) and also the best scores for the metrics, *S*, TE and FNR. It is somewhat outperformed by the *MoG_D_*, PBAS and SOM algorithms in the FPR metrics. However, BayesNet achieves again the best global results indicated by the *RM* and *S* metrics.

**Figure 5.**
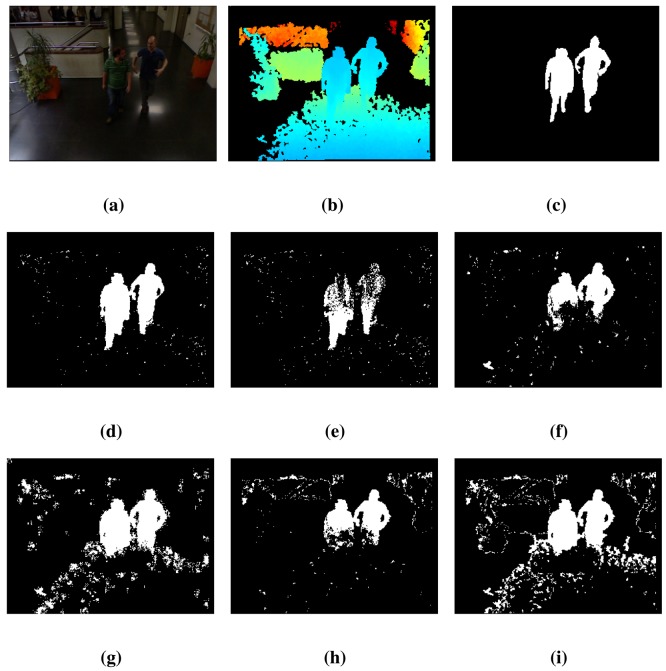
Results for frame 1, 069 of the *cam*1 sequence. See the text for a detailed explanation. (**a**) Color; (**b**) Depth; (**c**) Ground truth; (**d**) BayesNet; (**e**) *MoG_D_*; (**f**) PBAS; (**g**) Vibe; (**h**) SOM; (**i**) *MoG_Ziυ_*.

**Figure 6.**
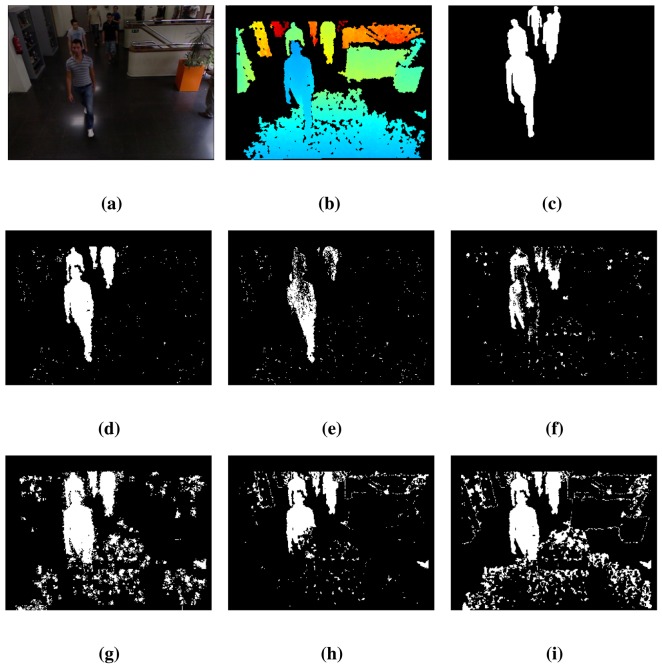
Results for frame 513 of the *cam*2 sequence. See the text for a detailed explanation. (**a**) Color; (**b**) Depth; (**c**) Ground truth; (**d**) BayesNet; (**e**) *MoG_D_*; (**f**) PBAS; (**g**) Vibe; (**h**) SOM; (**i**) *MoG_Ziυ_*.

Figures 5 and 6 show some qualitative FG/BG segmentation results for frame 1, 069 of the sequence, *cam*1, and for frame 513 of the sequence, *cam*2, respectively. The arrangement is the same as in Figure 3. Similarly to the other dataset, the images shows large areas for which the depth data is not available, due to reflective surfaces and out-of-range objects. Clearly, the proposed method, BayesNet, outperforms the others, achieving better accuracy in the segmentation and less false negatives.

Finally, Table 7 shows the ranking of all state-of-the-art methods considering all the sequences. The proposed technique, BayesNet, achieves the best score, which is mainly attributed to the spatial and depth dynamic models that explicitly exploit the inherent properties of the depth imagery. As can be noticed, the BayesNet approach always guarantees an improvement of performance with respect to the *MoG-BS* module, which is represented by the *MoG_D_* algorithm. It also outperforms the other state-of-the-art techniques in all the sequences, including the PBAS algorithm that has performed very well in various competitions for background subtraction algorithms (see, for example, the results presented in [34]). There is also a gap in performance between the BayesNet and *MoG_D_* algorithms and the others, which is mainly attributed to the adaptive processing of the Kinect sensor noise.

### 4.1. Operational and Practical Issues

In this subsection, several operational and practical issues are addressed, such as the computational cost, the relationship between the MoG-BS and BN-FBP modules, the robustness to several factors (missing and noisy depth measurements, camera jitter, intermittent motion and the viewpoint change of foreground objects) and the initialization.

The computational cost has been calculated as the mean value of the processing time of the algorithm using two different image sizes: 320 × 240 and 640 × 320 pixels. The computer used for the tests had an Intel Core i7-3540M processor at 3 GHz and 12 GB of RAM. The obtained mean values have been 432 ms and 1, 106 ms for the first and second image sizes, respectively. Notice that the algorithm is currently a prototype implemented in MATLAB without any specific code optimization, and therefore, the aforementioned processing times can be decreased by either optimizing the MATLAB-based implementation, programming a c/c++ implementation, or even programming a graphical processing unit (GPU) -based implementation. Of special interest is the last choice: the structure of the BN-FBP module allows for an efficient implementation in a GPU, because the inference is based on a particle filtering technique, in which the computation relative to each particle can be performed in parallel. The implementation of the other algorithms, *MoG_D_*, PBAS, Vibe, SOM and *MoG_Ziυ_*, is based on c/c++, and their corresponding processing times are 25.1, 73, 4.6, 7.8 and 3.5 ms, respectively, for images of a size of 320 × 240 and 95, 121, 18, 47.5 and 20 ms, respectively, for images of a size of 640 × 320.

Taking into account that the MoG-BS module is essentially the *MoG_D_* algorithm, the BN-FBP module is the most expensive in terms of computational cost. Nonetheless, both modules are essential to achieve a superior segmentation performance. The MoG algorithm models a potentially multi-modal background by a Mixture of Gaussian distribution and detects foreground pixels as those that do not fit in the background distribution. The MoG does not use the foreground dynamics to improve the segmentation, only the background dynamics (which is assumed to be static, quasi-static or with a repetitive/periodic motion). In addition, the foreground/background classification is performed at the pixel level, which is more sensitive to noise. On the contrary, the BN-FBP module takes explicitly into account the foreground dynamics in the three dimensions (width, height and depth) to improve the foreground segmentation. In addition, the foreground/background classification is performed at the region level, and therefore, it is more robust to noise. However, the Bayesian Network only considers the information given by two consecutive images, whereas the MoG recursively uses all the images. Thus, both methods complement each other.

Regarding the noisy depth measurements, the robustness is due to the region-level processing performed by the BN-FBP module. Working with regions instead of pixels allows for the consideration more data to make inferences and, thus, to be less sensitive to noise. Specifically, this behavior is achieved by working with histograms of depth regions, rather than individual pixel values. On the other hand, the quadratic relationship between the measured depth and the noise is taken into account in both system modules. In the MoG-BS module, the depth model parameters are selected as follows: given the mean value of each Gaussian of the mixture, its variance is adjusted according to the aforementioned quadratic relationship [22]. In the BN-FBP module, the quadratic relationship of the Kinect sensors is indirectly handled by the online logistic regression classifier. The training data used by the classifier are the predicted foreground and background depth histograms, which, in turn, have been obtained (via the spatial and depth dynamic models) from the noisy depth histograms of the FG/BG regions in the previous time step.

Due to the previous adapted noise processing, the operational range of the Kinect sensor can be extended further than the recommended operational conditions: from 7–8 m up to 11–12 m, which is usually not considered, because of the low signal-to-noise ratio.

Regarding the missing depth measurements, the region-level processing is also the key. Inside a region, there can be some pixels without depth assignment, but the characterization of such a region can be still done using the other pixel values. In addition, this region-level processing should theoretically provide a natural robustness against the camera jitter.

As regards the intermittent motion of background objects (dynamics backgrounds), the algorithm has not been explicitly designed to be robust to this situation, and therefore, a decrease in the performance is expected.

**Figure 7.**
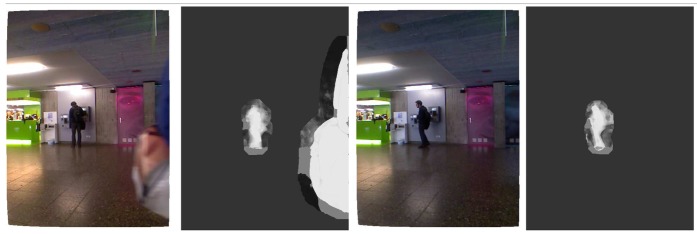
Posterior pdf of an articulated foreground object between two images without taking into account the background data/model. (**a**) color; (**b**) pdf of foreground; (**c**) color; (**d**) pdf of foreground.

The proposed algorithm is also robust to viewpoint changes of foreground objects thanks to the underlying piecewise-linear model used for the depth-based foreground dynamics in the BN-FBP module. Briefly, foreground regions in the previous time step are divided into sub-regions. For every sub-region, different possible depth displacements are calculated for the actual time step (as part of the particle filtering procedure). As a result, a bag' of possible local regions with different depth structures is available, which cover the actual depth appearance of the foreground, including potential deformable evolutions, such as articulated foreground objects or changes in the view point. This fact can be observed in Figure 7, where the posterior pdf of the foreground regions (*i.e.*, without taking into account the background data/model), computed by the BN-FBP module, is shown for two images. In spite of the articulated and abrupt motion of the human involved in the scene (a fast rotation), the estimated/predicted foreground regions (brighter regions) are quite accurate thanks to the spatial and depth dynamic models.

The process of initialization is explained below. The first frame (usually free of foreground objects) is used to initialize the probabilistic background model of the MoG algorithm (MoG-BS module). From the second frame on, the MoG-BS module is already able to compute FG/BG segmentations. On the other hand, the BN-FBP module needs the background model and the FG/BG segmentation from the previous time step to estimate the FG/BG segmentation in the current time step. All this data is already available from the third frame on thanks to the MoG-BS module. No more considerations are needed for the BN-FBP module, since it is not a temporal recursive model.

## 5. Conclusions

A novel algorithm for high-quality foreground segmentation in depth imagery has been proposed, which can operate almost independently of the existing illumination conditions in indoor scenarios. The FG/BG segmentation is carried out by the combination of a MoG-based subtraction algorithm and a Bayesian network-based algorithm. The Bayesian network is able to predict the FG/BG regions between consecutive depth images by explicitly exploiting the intrinsic characteristic of the depth data. For this purpose, two dynamic models that estimate the spatial and depth evolution of the FG/BG are used. Of special interest is the depth-based dynamic model that predicts the depth distribution of the FG/BG objects in consecutive time steps, which are encoded by an appearance model based on the concept of bag of features'. Remarkable results have been obtained in two public depth-based datasets, outperforming other state-of-the art approaches.

## Figures and Tables

**Table 1.** Summary of variables and main parameters used in the proposed Bayesian network.

Variable/Parameter	Description
*FG_t_*	FG/BG binary image segmentation at time step *t*
*FG_t_*(*x*)	Binary value of *FG_t_* at the pixel coordinates, *x*
*H_t_*	Set of depth histograms computed from *DI_t_*
*H_t_*(*x*)	Depth histogram computed from a local region of *DI_t_* centered on the pixel coordinates, *x*
*FGH_t_*	Set of depth histograms that models the foreground appearance at the time step, *t*
*FGH_t_*(*i*)	*i*–*th* depth histogram in *FGH_t_*
*ST*	Expected shift in depth of the foreground regions between consecutive time steps
*BGH_t_*	Set of depth histograms that models the background appearance at the time step, *t*
*BGH_t_*(*j*)	*j*–*th* depth histogram in *BGH_t_*
*DI_t_*	Depth image at time step *t*
*DBG_t_*	Depth-based representation of the most probable background obtained from the MoG-BS module at time step *t*

**Table 2.** Detection accuracy obtained by analyzing the *lobby*1 sequence. TE, total error; FNR, false negative rate; FPR, false positive rate; *MoG_D_*, depth-mixture-of-Gaussian foreground segmentation; PBAS, pixel-based adaptive segmenter; SOM, self-organizing map; *MoG_Ziυ_*, adaptive-Gaussian-mixture-model foreground segmentation.

	**TE**	**FNR**	**FPR**	***S***	***RM***
**BayesNet**	**3.14**	24.63	1.19	**0.48**	**1.5**
*MoG_D_*	3.65	31.70	**1.11**	0.34	2
PBAS	5.29	39.19	2.22	0.28	3.25
Vibe	33.36	**19.97**	34.57	0.14	4.75
SOM	13.32	40.39	10.86	0.19	4.75
*MoG_Ziυ_*,	6.02	41.31	2.82	0.19	4.75

**Table 3.** Detection accuracy obtained by analyzing the *lobby*2 sequence.

	**TE**	**FNR**	**FPR**	***S***	***RM***
**BayesNet**	**4.84**	26.30	1.76	**0.46**	**1.5**
*MoG_D_*	5.42	31.38	**1.69**	0.41	2.75
PBAS	6.53	29.30	3.26	0.43	3.25
Vibe	18.73	**22.06**	18.26	0.26	4.75
SOM	9.79	38.25	5.71	0.33	5.25
*MoG_Ziυ_*	6.51	27.43	3.51	0.39	3.50

**Table 4.** Detection accuracy obtained by analyzing the *lobby*3 sequence.

	**TE**	**FNR**	**FPR**	***S***	***RM***
**BayesNet**	**4.68**	33.21	0.98	**0.51**	**2.25**
*MoG_D_*	7.20	56.99	**0.76**	0.32	4.25
PBAS	6.01	32.03	2.64	**0.51**	**2.25**
Vibe	9.02	**17.82**	7.88	0.45	4.25
SOM	7.47	23.37	5.41	0.47	3.75
*MoG_Ziυ_*	7.05	38.82	2.94	0.42	4.25

**Table 5.** Detection accuracy obtained by analyzing the *cam*1 sequence.

	**TE**	**FNR**	**FPR**	***S***	***RM***
**BayesNet**	**7.36**	**4.54**	7.86	**0.63**	**1.75**
*MoG_D_*	7.97	12.01	7.35	0.59	2.75
PBAS	14.12	61.80	**6.16**	0.29	4.25
Vibe	19.27	6.19	21.47	0.39	4.75
SOM	13.32	49.93	7.21	0.36	3.75
*MoG_Ziυ_*	17.63	5.68	19.64	0.41	3.75

**Table 6.** Detection accuracy obtained by analyzing the *cam*2 sequence.

	**TE**	**FNR**	**FPR**	***S***	***RM***
**BayesNet**	**7.21**	**0.96**	8.13	**0.60**	**1.75**
*MoG_D_*	7.30	17.37	**5.98**	0.55	2.25
PBAS	12.31	55.02	6.45	0.32	4.75
Vibe	14.44	7.34	15.43	0.42	5.00
SOM	10.26	38.30	6.43	0.43	3.25
*MoG_Ziυ_*	13.49	5.33	14.59	0.43	4.00

**Table 7.** Final ranking.

	***RC***
**BayesNet**	**1.75**
*MoG_D_*	2.80
PBAS	3.55
Vibe	4.70
SOM	4.15
*MoG_Ziυ_*	4.05
